# Fungicide Difenoconazole Induced Biochemical and Developmental Toxicity in Wheat (*Triticum aestivum* L.)

**DOI:** 10.3390/plants10112304

**Published:** 2021-10-26

**Authors:** Runqiang Liu, Jingchong Li, Lei Zhang, Ta Feng, Zhiyong Zhang, Baohong Zhang

**Affiliations:** 1 Henan Key Laboratory for Molecular Ecology and Germplasm Innovation of Cotton and Wheat, Henan Collaborative Innovation Center of Modern Biological Breeding, School of Resources and Environment, Henan Institute of Science and Technology, Xinxiang 453003, China; liurunqiang@hist.edu.cn (R.L.); lijingchong1995@163.com (J.L.); zhang13939679215@163.com (L.Z.); 2 Shanxi Mei Bang Pharmaceutical Group Co., Ltd., Weinan 714000, China; fengta1986@126.com; 3 Department of Biology, East Carolina University, Greenville, NC 27858, USA

**Keywords:** fungicide, toxicity, oxidative stress, wheat

## Abstract

Difenoconazole is one of the most commonly used fungicides to prevent and treat plant diseases caused by certain fungi. Due to increasing usage, more difenoconazole has been released into the environment and caused environment pollution. However, the potential impact of difenoconazole on plant growth and development and its involved mechanism are unclear. In this study, we discovered that difenoconazole exposure significantly inhibited plant growth, evidenced by the decrease in root dry weight, total root length, and surface area by 20–70%, 43–73%, and 26–66%, respectively, under different regimes of treatment concentrations and periods. Difenoconazole exposure also significantly inhibited shoot growth and development by decreasing 33–61% of the shoot dry weight and 50–65% of the leaf area. Difenoconazole exposure induced plant leaf cells to generate more ROS (O_2_^•−^ and H_2_O_2_) and MDA, which resulted in a decreased chlorophyll content and then inhibited leaf photosynthesis. Difenoconazole exposure also induced the activities of superoxide dismutase (SOD), catalase (CAT), guaiacol peroxidase (G-POD), and ascorbate peroxidase (APX) in the roots and leaves of the wheat seedlings. SOD and APX activities were higher and more stable in the roots than those in the leaves. Based on our study, plant roots exhibited a more pronounced superoxide radical scavenging ability than plant leaves. In summary, difenoconazole exposure caused oxidative stress, reduced chlorophyll biosynthesis and functions, and then inhibited wheat plant growth and development.

## 1. Introduction

Both biotic and abiotic stresses, especially biotic stresses (e.g., pests, diseases, and weeds) [1], are important factors restricting plant growth and agricultural production. Among them, fungal diseases have caused a loss in global crop yields of nearly 20% [2]. The application of chemical fungicides has been considered the primary method to protect crops from numerous diseases due to their convenience and low cost [3].

Although the effects of fungicides are confirmed in controlling diseases and increasing crop yields, their toxic effects on crop plants have not been well studied, although some studies demonstrated that fungicides might affect plant respiration [4], secondary metabolites [5], the synthesis of plant hormones [6], the synthesis and degradation of chlorophyll [7], and photosynthesis [8].

Pesticides and herbicides were shown to induce oxidative stress in plants [9]. Fungicides, a type of pesticide, might also cause a change in reactive oxygen species (ROS) levels in plant cells. To repair the ROS-induced damage, plants have evolved a complicated antioxidant enzymatic system (e.g., superoxide dismutase (SOD), catalase (CAT), guaiacol peroxidase (G-POD), glutathione reductase (GR), and ascorbate peroxidase (APX)), which can efficiently maintain redox homeostasis in plant cells by scavenging excessive ROS [10].

Difenoconazole is a broad-spectrum triazole fungicide; it is the active ingredient of numerous commercial fungicides, which pertains to 14 α-demethylation inhibitors (DMIs) [11]. Additionally, difenoconazole could be splendidly absorbed by plant roots and delivered to other parts of plants via xylem tissue. The usage of difenoconazole in agriculture has been rising faster than that of other fungicides for its efficiency in diseases [12], and it is usually used by spraying or seed coating [13] as well as by directly applying it to roots [14]. However, existing studies largely focused on difenoconazole residue in soil/crops and its effect against diseases, while its phytotoxicity has rarely been reported.

Wheat is an important cereal crop and one of the main food crops in the world, which provides about 35% of the food energy for the world’s population. Thus, ensuring a high and stable wheat yield is of great significance to the world’s food security [15]. With the quick growth of the human population and the improvement in the economy, the demand for food and energy has increased rapidly, and the sustainable production of wheat has attracted increased attention.

Root application of fungicides can effectively prevent fungal diseases by preventing soil-borne fungi. To our best knowledge, although some studies have used difenoconazole for root application such as applying it to honeysuckle by root irrigation [14], its effect on plant growth and its mechanism of action remain unclear. The main aims of this study were as follows: (1) to investigate the effects of difenoconazole on root and shoot development of wheat plants as well as the accumulation of biomass, chlorophyll content, and chlorophyll a fluorescence characteristics, and then reveal the connections between plant morphology change and potential photosynthetic activities; (2) to demonstrate the relationship between the effects and the oxidative stress induced by difenoconazole by determining the ROS level, antioxidant enzyme activity, malondialdehyde (MDA) content, and electrolyte leakage for elucidating the potential mechanism of action of the effect of difenoconazole on wheat morphology and physiology.

## 2. Materials and Methods

### 2.1. Plant Materials and Culture Conditions

The major goal of this study was to investigate the effects of difenoconazole, uptaken by wheat roots, on the growth of whole seedling plants. However, since the growth of the roots is difficult to directly observe with soil culture, this study was conducted by hydroponics according to previously described methods [16,17].

Seeds of the wheat cultivar Bainong 207 (bred by the Wheat Genetic Improvement Research Center of Henan Institute of Science and Technology) were selected in this study. The seed treatment and planting procedures followed our previous reports [16,17]. Briefly, the wheat seeds were sterilized in 10% hydrogen peroxide (H_2_O_2_) for 10 min and then rinsed with distilled water 3–5 times to eliminate the H_2_O_2_ residues. Subsequently, the seeds were rolled into germination paper (30 seeds per roll) and then germinated in the dark at 28 °C, with a relative humidity of 75%. After 7 days of germination, seedlings with a uniform size were selected, and 16 seedlings were transplanted into plastic pots (25 cm in length, 20 cm in width, and 15 cm in height) with 5 L modified Hoagland nutrient solution. All wheat seedlings were cultivated under the same agronomic conditions (14 h light/10 h dark; the culture temperature was 30 ± 2 °C for the light stage, and 25 ± 2 °C for the dark stage). The modified Hoagland medium was supplemented with the following components: NaCl (2 mM), Ca(NO_3_)_2_ (2.5 mM), NH_4_H_2_PO_4_ (0.5 mM), MgSO_4_ (1 mM), EDTA-FeNa (0.1 mM), H_3_BO_3_ (0.02 mM), ZnSO_4_ (0.001 mM), MnSO_4_ (0.001 mM), CuSO_4_ (0.2 μM), and (NH_4_)_6_Mo_7_O_24_ (0.005 μM).

Four treatment concentrations of difenoconazole were used in this study. Based on a previous study [18], 100 mg/L was selected as the optimum concentration, which is the concentration applied in practical wheat production. In addition, a low concentration of difenoconazole (50 mg/L) and a high concentration (200 mg/L) were used, and 0 mg/L was used as the control. The transplanted wheat seedlings were first grown for 7 days under nutrient solution conditions and then moved to 5L Hoagland nutrient solutions supplemented with a range of concentrations (0, 50, 100, 200 mg/L) of difenoconazole. The treatments and the controls were arranged in a randomized block design with the respective treatment represented by five replicate pots. At 3 and 6 days after treatment (DAT), the wheat seedlings were collected, and the biomass, plant height, total root length, and area of leaves and roots were measured; the physiology parameters, including ROS level, MDA content, and antioxidant enzyme activity, were detected. Each treatment was run in three biological replicates, in which each replicate contained three to five plants.

### 2.2. Morphology Parameters and Biomass

Five seedlings were randomly selected from each treatment group and the control. The roots and leaves were separated from each selected seedling and then scanned by using the Epson Perfection V800 Photo scanner (Epson America lnc., Hillsboro, OR, USA). Subsequently, the WinRHIZO 2007 (Regent Instruments Inc., Quebec, QC, Canada) was adopted to investigate the morphological indicators, including the area of leaves and roots, the total root length, and the volume of roots. At the same time, each plant’s height was also measured. After these morphology measurements, the fresh weights of roots and shoots were weighed and recorded.

For plant dry weight, the above-mentioned shoots and roots were first dried in an electro-thermal blast drying oven (DHG-2200B, Zheng-118 Zhou Shengyuan Instrument Co., Ltd., Zhengzhou, China) at 105 °C for 30 min and then kept at 80 °C for 6 h before measuring their dry weight.

### 2.3. Chlorophyll Content and Chlorophyll a Fluorescence Characteristics

The second fully opened leaves of wheat seedlings were selected to measure the chlorophyll content and chlorophyll a fluorescence parameters. With the Dualex Scientific ^TM^ portable fluorometer (Force-A, Orsay, France), the chlorophyll (chl) content, flavonoids (Flav), and nitrogen balance index (NBI) were measured. The chlorophyll a fluorescence parameters, including maximum fluorescence (Fm) and minimal fluorescence (Fo), were detected with a chlorophyll fluorescence system (PEA, Hansatech Instruments Ltd., King’s Lynn, Norfolk, UK) after 30 min dark adaptation. The values of the maximal photochemical efficiency (Fv/Fm) and potential activity of PSII (Fv/Fo) were calculated as follows: Fv/Fm = (Fm − Fo)/Fm, and Fv/Fo = (Fm − Fo)/Fv.

### 2.4. Lipid Peroxidation and Relative Electrolytic Leakage

The level of malondialdehyde (MDA) reveals the degree of lipid peroxidation. It was detected by measuring the absorbance at 450 nm, 532 nm, and 600 nm of the red compound formed in the reaction between cellular MDA and thiobarbituric acid (TBA). Briefly, 0.5 g of samples was homogenized in 5 mL 10% (*w/v*) trichloroacetic acid (TCA). Subsequently, the suspension was centrifuged at 4000× *g* for 10 min. Then, 0.5 mL of supernatant was mixed with 0.5 mL of 0.6% TBA; the mixture was heated in boiling water for 20 min and then cooled immediately. Following that, the mixture was centrifuged at 10,000× *g* for 10 min, and the absorbance of the reaction was examined at 450 nm, 532 nm, and 600 nm. The MDA content was calculated as nmol g^−1^ fresh weight.

Relative electrolyte leakage was measured according to a previous report [19]. Briefly, 0.2 g root or leaf samples was cut into pieces of about 1 cm in length and then placed in a 15 mL centrifuge tube containing 10 mL of deionized water at 35 °C for 2 h. Then, the electrical conductivity of the solution (Lt) was measured. Subsequently, the samples were boiled in a water bath for 30 min, and the final electrical conductivity (L_0_) was measured after cooling to 25 °C. The relative electrolyte leakage was defined as (L_t_/L_0_) × 100.

### 2.5. Detection of ROS

ROS were determined by the O_2_^•−^ and H_2_O_2_ levels in roots and leaves. Superoxide anion (O_2_^•−^) levels were measured following a previous report [20]. Briefly, root and leaf samples were ground with liquid nitrogen, then 6.25 mM phosphate buffer was added, and the samples were centrifuged at 10,000× *g* at 4 °C for 20 min; the supernatants were collected to determine O_2_^•−^. The initial mixture (comprising 0.1 mL supernatant, 0.1 mL 50 mM phosphate buffer (pH 7.8), and 0.2 mL and 0.25 mM hydroxylamine hydrochloride) was incubated at 25 °C in the dark for 1 h. Then, 0.2 mL 4.25 mM p-aminobenzene sulphonic acid and 0.2 mL 1.75 mM α-naphthylamine were added. The final mixture of 0.8 mL was incubated at 25 °C in the dark for 20 min. The absorbance was examined at 530 nm and was used to calculate the O_2_^•−^ concentration. The O_2_^•−^ standards were prepared with NaNO_2_.

With a modified ferrous ammonium sulphate/xylenol orange (FOX) method, hydrogen peroxide (H_2_O_2_) levels were determined. The samples were ground with liquid nitrogen and extracted by cold acetone, and the supernatants were collected after being centrifuged at 10,000× *g* at 4 °C for 15 min. The reaction (1 mL) contained 0.15 mL supernatants, 0.1 mL distilled water, 0.25 mL 400 mM sorbitol, 0.25 mL 0.8 mM ferrous ammonium sulphate, and 0.25 mL 10.4 mM xylenol orange. After the reaction was incubated at 30 °C for 30 min, the absorbance was determined at 560 nm. The standards were prepared with diluted 30% H_2_O_2_.

### 2.6. Activities of Antioxidant Enzymes

Antioxidant enzymes were extracted and measured according to a previous report [21]. Briefly, 0.5 g of fresh leaf/root sample was ground into powder under liquid nitrogen and then suspended in 5 mL phosphate buffer (100 mM, pH 7.5) supplemented with 1% polyvinylpyrrolidone (PVP). The homogenates were centrifuged at 15,000× *g* at 4 °C for 20 min, and the supernatant was collected to determine enzyme activities.

SOD (EC 1.15.1.1) activity was determined using the nitroblue tetrazolium (NBT) light reduction method [22]. SOD inhibited the reaction between riboflavin and NBT, and the SOD activity was calculated by examining the absorbance of the reaction at 560 nm after 30 min of light. One unit of enzyme activity was defined as the amount of enzyme required to inhibit the reduction in NBT to half of the control.

G-POD (EC1.11.1.7) activity was measured according to a previous report [23]. Briefly, G-POD catalyzed the oxidation of guaiacol to produce brown substances, and the rate of increase in absorbance of the brown substances was determined for 3 min at 470 nm. The 2 mL reaction mixture contained 1.5 mL 1.92 mM guaiacol (dissolved in 100 mM phosphate buffer at pH 6.5), 0.4 mL 50 mM H_2_O_2_, and 0.1 mL enzyme extract.

CAT (EC1.11.1.6) activities were determined according to a method reported by Chen et al. [24]. The rate of change in H_2_O_2_ absorbance for 1 min at 240 nm was employed to determine the CAT activity. The 2 mL reaction mixture contained 0.7 mL 100 mM phosphate buffer (pH 7.0), 0.3 mL 100 mM H_2_O_2_, and 1 mL enzyme extract. The APX (EC1.11.1.11) activity was measured following the procedure of Koricheva et al. [25]. The APX activity was calculated by measuring the rate of decrease in absorbance of the reaction at 290 nm. The 2 mL reaction mixture contained 1.1 mL 0.75 mM ascorbate (dissolved in 50 mM phosphate buffer at pH 7.0), 0.5 mL H_2_O_2_, and 0.4 mL enzyme extract. GR activity (EC1.6.4.2) was measured based on the report by Mandhania et al. [26]. The decrease in absorbance was monitored at 340 nm. The 2 mL reaction mixture contained 0.9 mL 200 mM phosphate buffer (pH 7.5), 0.4 mL 2.5 mM GSSG, 0.4 mL 1 mM NADPH, and 0.3 mL enzyme extract.

The G-POD, CAT, APX, and GR activities were represented by the fresh weight (FW), and one unit of enzyme activity was defined as 0.01 unit of the OD value change per min, as expressed by unit mg^−1^ FW.

### 2.7. Statistical Analysis

The biomass and morphology were tested in quintuplicate, and the remaining tests were performed in triplicate. The results were statistically analyzed by analysis of variance (ANOVA). The differences between treatments were investigated by performing Duncan’s multiple range test at a *p* < 0.05 significance level. All statistical analyses were conducted with SPSS software (ver. 22.0; SPSS Inc., Chicago, IL, USA).

## 3. Results

### 3.1. Effects of Difenoconazole on the Growth of Wheat Seedlings

Difenoconazole significantly affected the growth of wheat seedlings. After the treatments with difenoconazole, the growth of wheat seedlings started to slow down compared with the controls. At 3 DAT, the seedlings were significantly different between the treatment groups and the controls, evidenced by the plant shape and size as well as the root development. At 6 DAT, the difference was more obvious between the treatment groups and the controls.

The fresh and dry weights of shoots and roots were measured at 3 and 6 DAT (Table 1). The root and shoot fresh weights of the treated seedlings were significantly (*p* < 0.05) lower than those of the controls, and the disparity between the control seedlings and the treated seedlings was anabatic with the increase in the difenoconazole concentrations and the increase in the treatment time. At 3 and 6 DAT, under the 200 mg/L treatment, the shoot fresh weight decreased by 57.6% and 73.3%, and the root fresh weight decreased by 60.5% and 69.9%, as compared with the controls, respectively. Unsurprisingly, difenoconazole also significantly affected the dry weight of the wheat seedlings (Table 1). At 3 DAT, compared with the control, the shoot dry weights of the seedlings treated with difenoconazole (50, 100, and 200 mg/L) decreased by 24.1%, 44.1%, and 46.6%, respectively, and the root dry weights decreased by 32.8%, 42.6%, and 51.4%, respectively. At 6 DAT, the differences were further enlarged in the dry weight between the seedlings of the control and the treatment groups, and the shoot and root dry weights were 69.6% and 67.8% lower than those of the controls under the 200 mg/L treatment, respectively.

With increasing treatment time from 3 to 6 DAT, the control fresh and dry plant weights increased by 69.6% and 113%, respectively. For the treatment groups, the maximum increment in the fresh and dry plant weights emerged at 50 and 100 mg/L, increasing by 21.2% and 38.7%, as compared with those at 3 DAT, respectively. Overall, the amount and rate of biomass accumulation were significantly lower in the treatment groups than those in the controls.

### 3.2. Effects of Difenoconazole on Root Development of Wheat Seedlings

The treatments with difenoconazole significantly affected the root development of wheat seedlings (Table 2). After the treatments, the total root length, the total root surface area, and the total root volume of seedlings were significantly lower than those of the controls (Table 2). The difference was enlarged between the treatment groups and the controls with increasing concentrations of difenoconazole and increasing treatment time. For example, compared with the controls, under the 200 mg/L treatment, the total root length, the total root surface area, and the total root volume of seedlings decreased by 38.9%, 35.4%, and 35.7% at 3 DAT, respectively, which declined by 70.1%, 66.7%, and 56.9% at 6 DAT in contrast, respectively. As the treatment time increased from 3 to 6 DAT, the total root length, the total root surface area, and the total root volume of the controls significantly increased, and those of the treatment groups only increased to a certain extent. However, according to the comparison between 6 and 3 DAT, the mentioned parameters (except for the total root volume) did not exhibit any obvious statistical difference among the three treatment groups.

The average root diameter displayed a different trend, which was incremental with the increase in the concentrations of difenoconazole. At 3 and 6 DAT, the average root diameter of the seedlings treated with 200 mg/L of difenoconazole was 23.7% and 20.6% higher than that in the controls.

### 3.3. Effects of Difenoconazole on Plant Height and Leaf Development of Wheat Seedlings

Difenoconazole can significantly affect leaf development. As indicated from the results of this study, the leaf area of the treated seedlings was significantly (*p* < 0.05) smaller than that of the controls. The difference was enlarged between the control seedlings and the treatment seedlings with the increase in the difenoconazole concentrations and the treatment time. At 3 DAT and 6 DAT, under the 200 mg/L difenoconazole treatment, the leaf area of seedlings was 58% and 61.9% less than that in the controls, respectively (Table 3). Additionally, under the normal condition, the plant leaves quickly developed. After 3 days of growth, the surface area increased from 32.26 to 43.81 cm^2^/plant, almost increasing by 35.8% (Table 3). Under the difenoconazole treatments, although the leaves were developed, the maximum increase in the total surface area of the leaves was only 26.4%.

The difenoconazole treatments also affected the plants’ height (Table 3). Before the treatments with difenoconazole, all plants’ heights were the same. At 3 DAT, the height of the control seedlings reached 26.68 cm, and the heights of the seedlings treated with 50, 100, and 200 mg/L of difenoconazole were only 20.70, 20.32, and 20.42 cm/plant, respectively. Upon increasing the treatment time from 3 to 6 DAT, all plants did not significantly vary compared with the results 3 days before, and the plant height of the controls increased by only 1.58 cm. For the treatment groups, the maximum increment in plant height was only 1.76 cm, which was found under the concentration of 50 mg/L.

### 3.4. Effects of Difenoconazole on the Chlorophyll Content and Chlorophyll a Florescence

The difenoconazole treatments led to an overall decrease in the Chl content and NBI (Table 4), and the magnitude of the reduction was enlarged with the increase in the concentrations of difenoconazole and treatment time. At 3 DAT, the Chl content and NBI significantly decreased in the seedlings treated with 50, 100, and 200 mg/L of difenoconazole; in the treatment groups, the Chl content decreased by 25.9%, 31.9%, and 37.5%, and the NBI decreased by 34.3%, 44.4%, and 50.9%, respectively. At 6 DAT, the Chl content and the NBI did not change in the control, and the Chl content of the treatment groups further decreased, which were 9.1%, 20.0%, and 5.7% lower than those at 3 DAT. However, the Flav content increased with the increase in the concentrations of difenoconazole and treatment time. At 3 DAT and 6 DAT, under the 200 mg/L difenoconazole treatment, the Flav content increased by 23.5% and 38.6% in comparison with the controls, respectively.

Treatment with difenoconazole also significantly affected the PSII activities (Table 4). Unsurprisingly, the Fv/Fm and Fv/Fo values displayed the same trends as the Chl content. The Fv/Fm and Fv/Fo values decreased with increasing concentrations of difenoconazole and increasing treatment time. At 3 DAT and 6 DAT, under the 200 mg/L difenoconazole treatment, the Fv/Fm values decreased by 8.0% and 9.9%, and Fv/Fo values decreased by 25.9% and 33.0%, in comparison with the controls, respectively.

### 3.5. Effects of Difenoconazole on Lipid Peroxidation and Electrolyte Leakage

**Figure 1:**
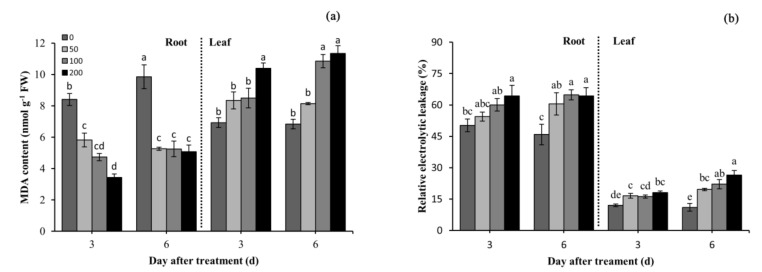
Effects of difenoconazole on MDA content (**a**) and relative electrolytic leakage (**b**) in leaves and roots of wheat seedlings. Different lowercase letters indicate significant difference (*p* < 0.05). Error bars indicate standard errors calculated for three replications. The 0, 50, 100, and 200 represent the different treatment concentrations.

The MDA level is used to indicate the degree of lipid peroxidation and the integrity of membranes. Difenoconazole significantly reduced the MDA content in the roots as compared with the controls (Figure 1a). At 3 DAT, the MDA contents decreased by 30.8%, 43.8%, and 59.1% in the roots of seedlings treated with 50, 100, and 200 mg/L of difenoconazole, respectively. At 6 DAT, the root MDA content was still significantly decreased in the treatment groups compared with the controls but was higher than that at 3 DAT (except for 50 mg/L). However, a converse trend of the MDA content in leaves was observed, and the MDA content augmented with the increased difenoconazole concentrations and the extension of the treatment time. At 3 DAT and 6 DAT, under the 200 mg/L difenoconazole treatment, the MDA content increased by 50.1% and 65.8% in contrast with the controls, respectively (Figure 1a). It is noteworthy that under the 100 and 200 mg/L difenoconazole treatments, the MDA content was higher at 6 DAT than that at 3 DAT.

The relative electrolytic leakage demonstrates the degree of cell membrane damage. The root and leaf electrolyte leakage of wheat seedlings was incremental with the increase in the difenoconazole concentrations (Figure 1b). At 3 DAT and 6 DAT, under the 200 mg/L difenoconazole treatment, the root electrolyte leakage was 28.1% and 43.1% higher than that in the controls, respectively. It is noteworthy that at 6 DAT, the root electron leakage almost did not change in the treatment groups compared with that at 3 DAT. However, except for the effect of the difenoconazole concentrations, the leaf electrolyte leakage was also affected by the increasing treatment time. At 6 DAT, the seedlings’ leaf electrolyte leakage was 18%, 36%, and 45% higher in the leaves treated with 50, 100, and 200 mg/L of difenoconazole than that at 3 DAT (Figure 1b). These results indicate that the degree of leaf cell membrane damage was incremental as the treatment time increased, but the degree of root cell membrane damage did not change.

### 3.6. Effects of Difenoconazole on ROS Biosynthesis

**Figure 2:**
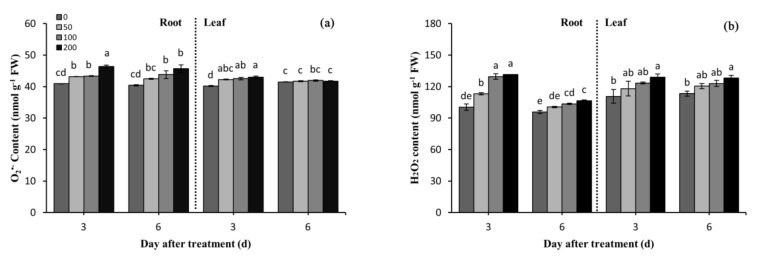
Effects of difenoconazole on O_2_^•−^ (**a**) and H_2_O_2_ (**b**) contents in leaves and roots of wheat seedlings. Different lowercase letters indicate significant difference (*p* < 0.05). Error bars indicate standard errors calculated for three replications. The 0, 50, 100, and 200 represent the different treatment concentrations.

Difenoconazole treatments led to significantly increased O_2_^•−^ levels in seedling roots compared with the controls, and its levels increased as the difenoconazole concentrations increased (Figure 2a). Under the 200 mg/L difenoconazole treatment, the O_2_^•−^ level increased in the roots by 13.3% at 3 DAT and 13.8% at 6 DAT as compared with the controls. However, after prolonging the treatment from 3 to 6 DAT, O_2_^•−^ levels did not significantly change. At 3 DAT, the leaf O_2_^•−^ levels were incremental with the increase in the difenoconazole concentrations, which increased by a maximum of 7.0% compared with the controls. However, there was not a significant difference in the leaf O_2_^•−^ levels at 6 DAT between the treatments and the controls.

Difenoconazole also led to a significant increase in H_2_O_2_ levels in the roots and leaves of the wheat seedlings, and the H_2_O_2_ levels increased as the difenoconazole concentrations increased. At 3 DAT and 6 DAT, under the 200 mg/L difenoconazole treatment, the H_2_O_2_ content increased by 30.9% and 11.1% in the roots, and the H_2_O_2_ content increased by 16.6% and 13.1% in the leaves, compared with the controls, respectively (Figure 2b). However, the H_2_O_2_ content decreased in the roots of the treatment groups but did not change in the shoots under difenoconazole treatments at 6 DAT compared with 3 DAT.

### 3.7. Effects of Difenoconazole on Antioxidant Enzyme Activities

**Figure 3:**
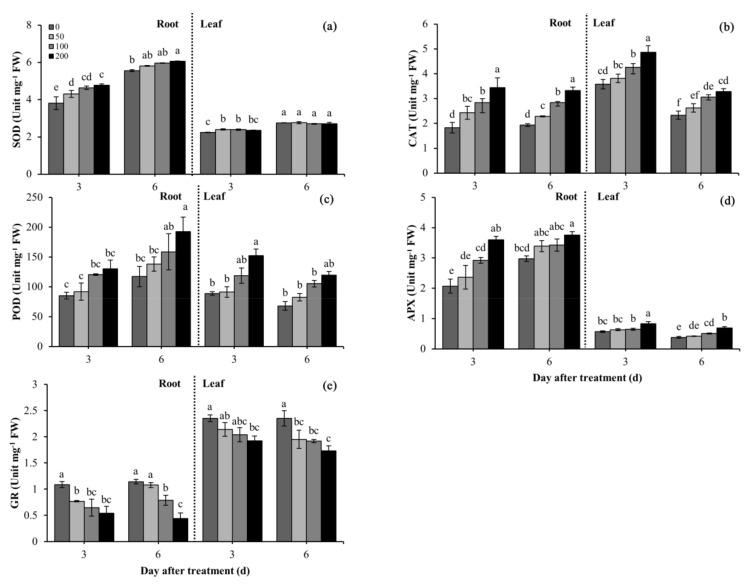
Effects of difenoconazole on superoxide dismutase (SOD) (**a**), catalase (CAT) (**b**), guaiacol peroxidase (G-POD) (**c**), ascorbate peroxidase (APX) (**d**), and glutathione reductase (GR) (**e**) activities in leaves and roots of wheat seedlings. Different lowercase letters indicate significant difference (*p* < 0.05). Error bars indicate standard errors calculated for three replications. The 0, 50, 100, and 200 represent the different treatment concentrations.

Difenoconazole significantly (*p* < 0.05) affected the activities of antioxidant enzymes, including SOD, CAT, G-POD, APX, and GR, particularly in the roots (Figure 3). SOD activities increased in the roots as the difenoconazole concentrations and treatment time increased. At 3 and 6 DAT, SOD activities increased by 25.4% and 9.0% in the roots under the 200 mg/L difenoconazole treatment in comparison with the controls, respectively (Figure 3a). At 3 DAT, SOD activities significantly increased in the leaves of the treatment groups in comparison with the controls, whereas no obvious difference was observed among the treatment groups, and the maximum increase was 7.0% with the 50 mg/L difenoconazole treatment. However, no statistical significance was reported in the control and the treatment groups at 6 DAT. It is noteworthy that SOD activities were higher in the roots than in the leaves.

Difenoconazole treatment also affected the activities of CAT, G-POD, and APX, and these changes increased as the difenoconazole concentrations increased (Figure 3b–d) At 3 DAT, under the 200 mg/L difenoconazole treatment, CAT, G-POD, and APX activities increased by 88.1%, 52.8%, and 73.9% in the roots, and by 36.0%, 71.4%, and 46.0% in the leaves, in comparison with the controls, respectively. At 6 DAT, under the 200 mg/L difenoconazole treatment, CAT, G-POD, and APX activities increased by 71.6%, 64.4%, and 26.2% in the roots, and by 11.4%, 76.1%, and 82.1% in leaves, in comparison with the controls, respectively (Figure 3b–d). Additionally, CAT, G-POD, and APX activities were lower in the leaves at 6 DAT than at 3 DAT, but G-POD and APX activities were slightly higher in the roots at 6 DAT than at 3 DAT.

Difenoconazole treatment significantly decreased GR activities; this decrease was enlarged as the difenoconazole concentrations increased (Figure 3e). At 3 DAT and 6 DAT, under the 200 mg/L difenoconazole treatment, GR activity decreased by 50.0% and 61.5% in the roots, and by 18.3% and 26.4% in the leaves, as compared with the controls, respectively. GR activities were higher in the roots at 6 DAT than at 3 DAT (except for the 200 mg/L difenoconazole treatment), but GR activities were slightly lower in the leaves at 6 DAT than at 3 DAT. Additionally, GR activities were higher in the leaves than in the roots across all treatment stages.

## 4. Discussion

In recent decades, fungicides have become crucial to prevent and control plant diseases in agricultural production. Although the effects of insecticides and herbicides on plant growth and development have been well studied at different levels, few studies have focused on the effects of fungicides on crop plants, and there is no report on the phytotoxicity of difenoconazole in wheat. Thus, it is very important to understand the physiological regulation mechanism of difenoconazole in wheat.

### 4.1. Growth Inhibition in Relation to Oxidative Stress

In this study, the phytotoxicity of difenoconazole was examined during wheat seedling stages. As indicated from the results, the exposure of difenoconazole significantly inhibited the growth of wheat seedlings (Table 1, Table 2, Table 3 and Table 4). Such inhibition in growth might be associated with difenoconazole accumulation in wheat plants that caused oxidative stress by inducing ROS accumulation in the cells. As a result, the chlorophyll content decreased, and the photosynthetic activities further decreased. As reported by Zhang et al. [27], use of the fungicide tebuconazolet could lead to a reduction in mesocotyl length in maize. The chlorophyll content and chlorophyll fluorescence parameters were negatively affected due to the phytotoxicity of difenoconazole (Table 4). Photosynthetic pigments act as vital factors for assessing plant responses to environmental stress, which directly determine photosynthesis efficiency. According to two previous reports [28,29], the fungicides fludioxonil and carbendazim considerably reduced the chlorophyll a, chlorophyll b, carotenoid, and total pigment contents in *Vitis vinifera* and *Nicotiana tabacum* plants. Since plants rely on photosynthesis for carbon assimilation to maintain growth and overall vitality, weakening photosynthesis could adversely affect plant growth and final biomass.

The NBI significantly decreased in the leaves due to the exposure to difenoconazole (Table 4). Such a reduction in the contents of nutrients was related to the inhibition of root growth (evidenced by the reductions in root length, surface, and volume) (Table 2). The vulnerable roots led to a reduction in the absorption of nutrients from the medium, which, in turn, affected the accumulation of nutrients in the leaves, caused a lower chlorophyll content, and inhibited shoot and root growth. Thus, this mutual inhibition forms a vicious circle, which seriously affects the normal growth of wheat seedlings.

The levels of H_2_O_2_ and O_2_^•−^, leaf MDA content, and electrolyte leakage increased significantly during the difenoconazole treatments (Figure 1a and Figure 2b). In addition, O_2_^•−^ levels increased in the treated roots at 3 DAT as compared with the controls, whereas O_2_^•−^ levels were not altered in the leaves among groups at 6 DAT. This demonstrates that H_2_O_2_ is critical to induce oxidative damage at 6 DAT. Biotic and abiotic stresses inhibiting plant growth are attributed to oxidative stress induced by ROS. ROS can react rapidly to almost all structural and functional organic molecules (e.g., proteins, lipids, and nucleic acids), thereby causing cell metabolic disorder and eventually inhibiting cell growth [30]. Similar types of fungicide-induced phytotoxic impacts on plant growth and overproduction of ROS have been reported in other plant species [7,31]. Difenoconazole induced ROS levels, which was probably because it exerted a toxic effect on plants after entering the wheat seedlings, consistent with the toxic effects of chlorpyrifos and imidacloprid on rice plants [32]. Although difenoconazole significantly elevated the levels of ROS, it led to different accumulations of MDA in the roots and leaves (the MDA content in the leaves increased significantly, whereas the content significantly decreased in the roots, in comparison with the controls) (Figure 2). This therefore suggests that difenoconazole caused oxidative damage to lipids and constituted the oxidative stress of the wheat seedling leaves, which further constituted cell membrane damage and increased electrolytic leakage. Additionally, the leaf MDA content and electrolytic leakage increased as the treatment times were prolonged. These phenomena indicate that the integrity of the cell membrane structure was further damaged, which supported the further decrease in the chlorophyll content at 6 DAT. Inevitably, the decrease in the chlorophyll content led to a decreased photosynthesis efficiency and the following inhibition in the growth of the wheat seedlings.

The decrease in the root MDA content in response to difenoconazole exposure indicates that the membrane integrity of the root cells was not affected (Figure 1a). A previous study reported varying decreases in the MDA content in soybean roots after both 24 and 72 h of glyphosate treatments [33], which is consistent with our results. This finding suggests that treatment with pesticides, including herbicides and fungicides, may lead to a reduction in the MDA content in the roots for a short time after treatment. However, the increased electrolytic leakage suggests that the root membrane integrity was damaged under the exposure to difenoconazole. It is noteworthy that the electrolytic leakage of all treatment groups did not obviously change at 6 DAT compared with that at 3 DAT, which demonstrates that the membrane damage was not further worsened, and not recovered either (Figure 1b). This might be explained by the following: (1) rapid increases in antioxidant enzyme activities during this period, thereby causing the redox balance of the root system to reach a new stable state to inhibit ROS-induced oxidative stress to the cell membrane; (2) at 3 DAT, the root system of the wheat seedlings adapted to the novel environment and exhibited the capacity to counteract the difenoconazole stress.

In fluorimetry, the ratio of variable fluorescence (Fv) to minimum fluorescence (Fo) (Fv/Fo) represents photosystem II (PSII) activity, and the ratio of Fv to maximum fluorescence (Fm) (Fv/Fm) represents the maximum efficiency of PSII photochemistry [34]. As demonstrated in the analysis of several chlorophyll a fluorescence parameters of plants after the treatment with fungicides [3,35,36], the values of Fm/Fv and Fv/Fo can be sensitive to oxidative stress. In this study, the Fv/Fm and Fv/Fo values significantly decreased under the exposure to difenoconazole (Table 4). As reported by Nason et al. [36], the application of strobilurin fungicides (picoxystrobin and pyraclostrobin) reduced the chlorophyll fluorescence parameter Fv/Fm in soybean seedling leaves. Such a reduction was attributed to the decrease in photochemical quenching (qP). Accordingly, O_2_^•−^ production in chloroplasts would increase as more electrons flow to O_2_. The excessive O_2_^•−^ and consequent H_2_O_2_ would damage the chloroplast membrane.

Plants respond to various stresses by accumulating metabolites including amino acids and flavonoids [37]. At the same time, flavonoids, including anthocyanin, flavone, flavonol, and isoflavone, are capable of resisting oxidative damage and improving plant stress resistance [38]. As proven by the increase in flavonoid accumulation in the fungicide-treated seedlings, fungicides may cause stress conditions consistent with those attributed to environmental aberrations. Notably, the Flav content was significantly induced by difenoconazole (Table 4), indicating the wheat plants positively responded to the difenoconazole treatment. In this study, although the wheat plants positively responded to difenoconazole exposure, they were still subjected to oxidative stress, and growth and development were retarded in the roots and shoots.

### 4.2. Difenoconazole Affects the Balance of Oxidation–Reduction

Oxidative stress refers to a result of ROS production over than elimination. To reduce and repair the damage associated with ROS, plants have evolved excellent antioxidant systems. Among plants’ antioxidant enzyme defense system, SOD plays a major role in catalyzing the disproportionation of O_2_^•−^ and converting O_2_^•−^ to O_2_ and H_2_O_2_. Increased SOD activity was reported in rice and wheat after herbicide treatments [39,40] as well as in vigna under insecticide exposure [41]. As expected, the seedlings treated with the fungicide difenoconazole showed significantly higher SOD activity than the controls, especially the activity of SOD in the wheat roots (Figure 3a). The increase in SOD activity could effectively convert O_2_^•−^ to O_2_ and H_2_O_2_ and avoid its excessive accumulation in wheat, as manifested by the O_2_^•−^ levels in the roots at 6 DAT, which were lower than those at 3 DAT. The consistency of H_2_O_2_ and SOD activity not only suggests that SOD could eliminate excessive O_2_^•−^ in the leaves but also confirms that oxidative damage was induced in the leaves by H_2_O_2_.

G-POD, CAT, and APX play an important role in the breakdown and removal of H_2_O_2_. A previous study showed that tomato *(Solanum lycopersicum Mill.)* plants increased the activities of G-POD, CAT, and APX during exposure to the fungicide thiram [7]. Our results also show that the activities of G-POD, CAT, and APX significantly increased during exposure of the wheat plants to difenoconazole (Figure 3b,c). This suggests that in the process of removing excessive H_2_O_2_, three antioxidant enzymes have a synergistic effect. Correspondingly, as indicated from the results of this study, the increase in the activities of G-POD, CAT, and APX in the roots could effectively detoxify H_2_O_2_, as demonstrated by the root H_2_O_2_ levels being significantly lower at 6 DAT than at 3 DAT (Figure 2b). However, this effect might be plant tissue, stress time, and stress degree dependent. As indicated from the results of this study, the activities of SOD, G-POD, and APX were significantly higher in the roots than in the leaves (Figure 3a,c,d), which may explain the decrease in the MDA content associated with plant exposure to difenoconazole in the roots (Figure 2). On the other hand, the induced CAT, SOD, and APX activities were insufficient to remove excessive H_2_O_2_ in the leaves (Figure 1b). The excessive accumulation of H_2_O_2_ was the main reason for the induced oxidative damage in the leaves. Additionally, as suggested from the results, the APX activity was 4-fold lower in the leaves than in the roots, and no significant difference was observed in CAT and SOD activities between the roots and leaves (Figure 3). The low leaf APX activity might be the main reason for why H_2_O_2_ could not be removed in the leaves in time.

Similar to APX, GR is one of the main components of the AsA-GSH cycle, and GR ensures the effective circulation of glutathione [41]. However, the treatment with difenoconazole significantly reduced GR activity in both the leaves and roots (Figure 3e). The inhibition of GR activity might lead to a decrease in the cycle rate of AsA-GSH and reduce the detoxification rate of ROS. The increased activities of three antioxidant enzymes (CAT, G-POD, and APX) could compensate for the lower GR activity in the roots, thereby avoiding excessive H_2_O_2_ accumulation in the cells. However, since the APX activities were lower in the leaves than in the roots, the negative effect of the decreased GR activities was magnified in the leaves. As a result, the excessive production of H_2_O_2_ in the leaves could not be removed in time. Moreover, this might be a unique toxic effect of the fungicide difenoconazole on plants.

## 5. Conclusions

Difenoconazole treatment significantly facilitated antioxidant enzyme activities (except for GR), but enzyme activities were not sufficiently boosted to scavenge excessive ROS, which then caused oxidative stress in the leaves. As a result, the chlorophyll content and Fv/Fm were reduced, and the growth and development of the seedlings were ultimately inhibited. These physiological results could be explained as the tolerance mechanism of plants and might help formulate countermeasures to reduce the risk of fungicide contamination in crop production. Moreover, subsequent research should be conducted to confirm the specific reasons for the decrease in the MDA content in the roots when being exposed to difenoconazole and its possible regulatory mechanism. Investigating the gene function using, for example, CRISPR/Cas9 genome editing [42,43] and gene expression analysis will help to elucidate the molecular mechanism.

## Figures and Tables

**Table 1:** Effect of difenoconazole on plant biomass of wheat seedlings ^Z^.

DAT * (Days)	Treatment (mg/L)	Fresh Weight (mg)			Dry Weight (mg)		
		Root	Shoot	Total Weight	Root	Shoot	Total Weight
3	0	1388.3 ± 31.4 b	1154.0 ± 20.1 b	2542.3 ± 28.1 b	98.7 ± 1.9 b	135.3 ± 7.9 b	234.0 ± 6.1 b
	50	805.0 ± 18.7 d	591.3 ± 10.7 d	1396.3 ± 29.4 d	67.3 ± 3.9 d	102.7 ± 4.3 c	170.0 ± 5.3 d
	100	679.3 ± 5.2 e	523.0 ± 9.8 ef	1202.3 ± 9.1 e	56.7 ± 1.8 e	75.7 ± 1.8 d	132.3 ± 1.0 f
	200	548.0 ± 6.1 f	489.5 ± 18.2 f	1037.5 ± 21.1 f	48.0 ± 1.5 f	72.3 ± 0.7 d	120.3 ± 1.0 f
6	0	2142.7 ± 33.7 a	2168.7 ± 51.3 a	4311.3 ± 79.8 a	169.0 ± 4.0 a	329.7 ± 10.9 a	498.7 ± 10.3 a
	50	971.3 ± 35.0 c	721.0 ± 4.2 c	1692.3 ± 33.8 c	81.3 ± 3.3 c	131.3 ± 8.7 b	212.7 ± 6.7 c
	100	771.3 ± 17.9 d	677.0 ± 7.1 c	1448.3 ± 22.3 d	70.0 ± 3.2 d	112.7 ± 3.0 c	182.7 ± 2.0 d
	200	645.5 ± 3.9 e	579.8 ± 7.2 de	1225.2 ± 6.5 e	54.5 ± 1.0 ef	100.2 ± 1.6 c	154.8 ± 1.5 e

^Z^ Values represent the mean ± standard error for each parameter. Different lowercase letters indicate significant difference (*p* < 0.05). * DAT: days after treatment.

**Table 2:** Effect of difenoconazole on root development of wheat seedlings ^Z^.

DAT * (Days)	Treatment (mg/L)	Total Root Length (cm)	Total Root Surface Area (cm^2^)	Total Root Volume (cm^3^)	Average Root Diameter (mm)
3	0	1266.86 ± 45.63 b	115.75 ± 2.71 b	0.84 ± 0.04 bc	0.29 ± 0.01 de
	50	847.20 ± 27.29 c	93.71 ± 4.10 bc	0.74 ± 0.04 c	0.35 ± 0.01 b
	100	811.49 ± 13.11 c	79.48 ± 2.11 c	0.68 ± 0.06 cd	0.36 ± 0.01 ab
	200	774.37 ± 15.24 c	74.82 ± 3.38 c	0.54 ± 0.05 d	0.38 ± 0.01 a
6	0	2857.22 ± 211.82 a	240.79 ± 20.77 a	1.74 ± 0.13 a	0.27 ± 0.01 e
	50	1111.28 ± 34.66 bc	106.70 ± 5.47 bc	0.93 ± 0.02 b	0.30 ± 0.00 cd
	100	939.24 ± 89.24 bc	89.84 ± 8.46 bc	0.82 ± 0.03 bc	0.33 ± 0.01 bc
	200	854.92 ± 142.46 c	80.35 ± 2.11 c	0.75 ± 0.02 c	0.34 ± 0.01 b

^Z^ Values represent the mean ± standard error for each parameter. Different lowercase letters indicate significant difference (*p* < 0.05). * DAT: days after treatment.

**Table 3:** Effect of difenoconazole on plant height and leaf development of wheat seedlings ^Z^.

DAT * (Days)	Treatment (mg/L)	Leaf Area (cm^2^)	Plant Height (cm)
3	0	32.26 ± 1.70 b	26.68 ± 1.74 a
	50	16.03 ± 1.01 de	20.70 ± 0.63 b
	100	14.18 ± 0.60 de	20.32 ± 0.44 b
	200	13.56 ± 0.59 e	20.42 ± 0.25 b
6	0	43.81 ± 0.69 a	28.26 ± 0.67 a
	50	20.66 ± 0.65 c	22.46 ± 0.42 b
	100	17.41 ± 0.69 d	21.30 ± 0.38 b
	200	16.68 ± 0.32 de	21.68 ± 0.54 b

^Z^ Values represent the mean ± standard error for each parameter. Different lowercase letters indicate significant difference (*p* < 0.05). * DAT: days after treatment.

**Table 4:** Effect of difenoconazole on chlorophyll content and fluorescence characteristics of wheat seedlings ^Z^.

DAT * (Days)	Treatment (mg/L)	Chl (μg/cm^−2^)	Flav (Dualex Units)	NBI (Dualex Units)	Fv/Fm	Fv/Fo
3	0	41.19 ± 1.56 a	0.79 ± 0.05 e	53.01 ± 2.22 a	0.81 ± 0.00 a	4.03 ± 0.23 a
	50	30.54 ± 1.32 b	0.89 ± 0.03 de	34.90 ± 2.36 b	0.77 ± 0.01 b	3.40 ± 0.25 b
	100	28.08 ± 1.92 bc	0.97 ± 0.03 cd	29.48 ± 2.48 bc	0.75 ± 0.02 bc	3.12 ± 0.16 bc
	200	25.75 ± 0.65 bcd	1.02 ± 0.05 bc	26.01 ± 1.80 cd	0.76 ± 0.01 bc	3.20 ± 0.12 bc
6	0	41.88 ± 0.94 a	0.90 ± 0.02 d	47.78 ± 2.09 a	0.81 ± 0.00 a	4.18 ± 0.06 a
	50	27.76 ± 3.48 bc	1.09 ± 0.03 ab	22.97 ± 1.32 d	0.77 ± 0.00 b	3.33 ± 0.06 bc
	100	22.38 ± 1.48 d	1.11 ± 0.03 ab	20.41 ± 1.68 d	0.76 ± 0.01 bc	3.12 ± 0.16 bc
	200	24.29 ± 1.30 cd	1.17 ± 0.04 a	20.88 ± 1.19 d	0.73 ± 0.02 c	2.80 ± 0.23 c

^Z^ Values represent the mean ± standard error for each parameter. Different lowercase letters indicate significant difference (*p* < 0.05). * DAT: days after treatment.

## Data Availability

Some or all of the data used during this study are available from the corresponding author upon request.
